# 

**Published:** 1846-01-01

**Authors:** 


AUSTIN FLINT, M. D.

Vol. 1. BUFFALO, JANUARY, 1846. No. 8.

### CONTENTS:

ORIGINAL COMMUNICATIONS.

Notes of an European Tour. By F. H. HAMILTON, M. D., Prof. of Surgery in Geneva Medical College. P. 170
Medical Quackery. By WM. TREAT, M. D. 173
Letter from Accoucheur. No. 4. 176
Needle found in the heart of a Cow. By J. H. BEECH, of Gaines, N. Y. 177
Distinctive characters of Remittent, Typhoid, and Typhus Fevers. By the Editor. 178

EDITORIAL, MEDICAL INTELLIGENCE, ETC.

Water Cure—Confessions and observations of Sir Edward Lytton Bulwer. 183
Summary of transactions of the College of Physicians of Philadelphia, from May to October, 1845, inclusive. 187
Introductory Lecture. By Walter Channing, M. D. 189
The People of the State of New-York, vs. Wm. B. Waterman. 190
Iodide of Iron. 191
On the nature of the Green Alvine evacuations of Children. 191
Dr. Pratt's artificial Nipple. 192
The Therapeutic influence of heated atmosphere. 192
Petrified Human Body. 192

BUFFALO:
JEWETT, THOMAS & CO., PRINTERS AND PUBLISHERS,
Exchange Buildings, Third Story, Main-Street.

